# Microbiota composition-based donor selection affects FMT efficacy in a murine colitis model

**DOI:** 10.3389/fimmu.2025.1635244

**Published:** 2025-08-01

**Authors:** Zhongming Dai, Wen Cheng, Huan Peng, Xiaokui Qiu, Jiawen Sun, Xiaoqiang Liu, Xianjiu Sun, Jinwei Cai, Jincui Wang, Guolong Li, Yongling Lv, Shaobo Chen, Zhongying Zhong

**Affiliations:** ^1^ Department of Gastroenterology, Qiu District People’s Hospital, Shenzhen, Shenzhen, Guangdong, China; ^2^ Department of Gastroenterology, Shenzhen Guangming District People's Hospital, Shenzhen, Guangdong, China

**Keywords:** gut microbiota, donor screening, FMT, IBD, 16S rRNA

## Abstract

**Background:**

Growing evidence links gut microbial dysbiosis to inflammatory bowel disease (IBD) pathogenesis, establishing fecal microbiota transplantation (FMT) as a microbiota-targeted therapy; however, variable outcomes in randomized trials highlight the need to identify compositional features of donor microbiota associated with FMT efficacy.

**Objective:**

This study aimed to investigate how the composition of the donor gut microbiota influences the therapeutic efficacy of FMT in IBD.

**Method:**

Fecal DNA from 39 IBD patients and 42 healthy donors was analyzed via 16S rRNA sequencing. Donor-enriched genera (identified through differential analysis and median abundance thresholds) guided FMT selection. Dextran sulfate sodium (DSS)-induced colitis mice received donor microbiota transplants; disease activity and microbiota dynamics were evaluated through longitudinal sequencing.

**Results:**

IBD patients showed reduced microbial diversity and increased Proteobacteria phylum versus healthy donors, as well as the genera *Escherichia-Shigella*, *Megamonas*, and *Klebsiella*. Linear discriminant analysis effect size (LEfSe) analysis identified 50 differentially abundant genera, with 36 beneficial taxa enriched in donors. Based on median abundance of these health-associated genera, four high- and low-abundance donors were selected. FMT from high-abundance donors outperformed low-abundance donors and 5-ASA in colitis mice, restoring microbial diversity to healthy levels. Recipient mice showed increased Firmicutes and Bacteroidota and decreased Verrucomicrobiota, with *Lactobacillus* and *Dubosiella* enrichment and normalization of *Lachnospiraceae NK4A136 group*, *Akkermansia*, *Turicibacter*, and *Parabacteroides*. LEfSe identified 24 genera distinguishing IBD and control mice; post-FMT microbiota of high-abundance donor recipients more closely resembled controls, correlating with therapeutic success.

**Conclusion:**

FMT ameliorated IBD symptoms in murine models, with therapeutic efficacy associated with the relative abundance of health-associated microbial genera in donor microbiota.

## Highlights

This investigation pioneers a composition-based donor stratification framework for FMT in IBD management, demonstrating three key methodological advancements:

- Moving beyond conventional single-strain supplementation paradigms to emphasize the ecological integrity and genus-level diversity of donor microbial communities;
- Establishing a systematic multi-donor comparison framework that identifies health-associated bacterial assemblages correlated with improved outcomes in murine colitis models;
- Revealing distinct compositional remodeling effects on host gut microbiota following FMT from donors with contrasting microbial profiles.

## Introduction

Inflammatory bowel disease (IBD), encompassing ulcerative colitis (UC) and Crohn’s disease (CD), represents a chronic relapsing-remitting inflammatory disorder of the gastrointestinal tract. Its global prevalence exhibits an escalating trajectory, particularly in emerging industrialized regions (1). The Global Burden of Disease study (2019) estimated 4.9 million prevalent cases worldwide, with notable demographic shifts toward earlier disease onset (2, 3). First-line pharmacological approaches include aminosalicylates, immunomodulators, and biologic agents (4). Although conventional therapies offer temporary symptom relief, prolonged use often leads to reduced efficacy and increased risk of disease progression (5). Surgical intervention remains a cornerstone of IBD management. Postoperative outcomes analysis reveals an 85% endoscopic recurrence rate within three years following ileocolonic resection in CD patients (6). Perioperative risks persist, with systematic reviews demonstrating 18% postoperative infection rates among surgically treated IBD population (7). These therapeutic challenges underscore the imperative for innovative treatment paradigms.

Accumulating evidence highlights gut microbial dysbiosis as a critical contributor to IBD pathogenesis (8, 9). In healthy individuals, the intestinal microbiota is predominantly composed of Firmicutes and Bacteroidetes phyla, constituting over 90% of the total microbial community (10). These commensal organisms maintain intestinal homeostasis through multifaceted mechanisms, including the production of short-chain fatty acids (SCFAs) and bile acids essential for epithelial barrier integrity and immune regulation (11, 12); and modulation of mucosal immunity via antimicrobial peptide (AMP) synthesis and immunoglobulin induction (10, 13). In contrast, IBD patients demonstrate distinct microbial perturbations marked by diminished populations of *Clostridium coccoides*; *Erysipelotrichales*; *Faecalibacterium prausnitzii* and *Bacteroidales*, concurrent with Enterobacteriaceae expansion (14–16). These alterations trigger pathophysiological cascades through impaired short-chain fatty acid (SCFA) biosynthesis, dysregulated bile acid metabolism, and compromised mucosal barrier integrity. Mechanistically, microbial metabolites exert direct immunomodulatory effects: propionate attenuates IL-17 secretion in colonic γδ T cells, while SCFA insufficiency activates NOD-like receptor (NLR) signaling pathways, thereby amplifying inflammatory cascades (17, 18). Preclinical evidence suggests the microbiota-bile acid axis may constitute a therapeutic target, with bile acid receptor modulation demonstrating anti-inflammatory effects in murine colitis models (19). Crucially, clinical disease severity exhibits positive correlation with dysbiosis magnitude, positioning microbiota-directed therapeutics as a rational intervention strategy.

Microbiota-targeted therapeutic strategies have emerged as a promising frontier in IBD management. FMT, a protocolized intervention for gut microbiome restoration, has demonstrated particular potential in reestablishing microbial homeostasis (20). The established efficacy of FMT in recurrent Clostridioides difficile infection (rCDI) treatment (achieving cure rates of 80-90%) has catalyzed its exploration for IBD therapeutics (21). Murine DSS-colitis studies reveal FMT-mediated amelioration of intestinal inflammation through STING pathway modulation, with concomitant restoration of microbial metabolic functions (22, 23). Clinical trials demonstrate FMT-induced microbiome diversification in IBD patients, reporting symptomatic improvement in approximately 83% of treated subjects across select cohorts, though therapeutic outcomes exhibit significant donor-dependent variability (24–26). Systematic pooling of clinical trial data reveals enhanced clinical remission rates associated with multi-donor, high-frequency FMT protocols when contrasted with single-dose administration strategies across IBD phenotypes (27). Collectively, these findings underscore that donor microbiota composition and ecological features may influence FMT efficacy, as suggested by preclinical and limited clinical observations. While much of the literature underscores the role of microbial dysbiosis in IBD, the specific contributions of donor microbiota to FMT outcomes remain poorly understood. The mechanisms and translatability of such findings to human donor screening remain to be clarified.

In this research, we initially examined the differences in microbiota between patients with IBD and healthy individuals using 16S rRNA sequencing and LEfSe analysis. We identified genera that were significantly more abundant in healthy populations and ranked the donors according to the median relative abundance of these genera. A mouse model of colitis induced by DSS was utilized to transplant the top four (high-abundance microbiota donors) and bottom four (low-abundance microbiota donors) ranked healthy human fecal microbiota, and we assessed the therapeutic effects of the various microbiota. The purpose of this study was to investigate how different composition of donor microbiota influence the effectiveness of FMT.

## Materials and methods

### Subject recruitment

This study enrolled 39 inflammatory bowel disease (IBD group) patients and 42 age-matched healthy controls (HC group) (age range: 20–70 years) from the Department of Gastroenterology at Shenzhen Guangming District People’s Hospital. The study protocol received ethical approval from the Institutional Review Board of Shenzhen Guangming District People’s Hospital (Approval No. LL-KT-2025006), with written informed consent obtained from all participants in accordance with the Declaration of Helsinki. IBD participants met the following inclusion criteria: (1) Diagnosis confirmed through standard clinical evaluation; (2) No familial history of gastrointestinal disorders; (3) Absence of antibiotic or immunomodulatory therapy within 30 days prior to enrollment. Exclusion criteria included: pregnancy, lactation, gastrointestinal surgery, other autoimmune diseases, and severe allergies. All IBD patients enrolled in the study were in an clinical remission state at the time of sample collection.

Healthy controls were required to: (1) Have no active medical conditions; (2) Refrain from antibiotic/immunotherapy use for 30 days pre-enrollment. Exclusion criteria for all participants included pregnancy, lactation, immunodeficiency disorders, and severe allergies. All patients were asked to follow their regular diet before data and sample collection.

### Sample collection

Fecal specimens were collected preprandially using sterile, gas-impermeable anaerobic collection tubes (Oxoid AnaeroGen, Thermo Fisher, USA) that were pre-filled with anaerobic gas-generating sachets to create an oxygen-free environment. The samples were immediately sealed and transported on ice to the laboratory within 30 minutes. Upon arrival, the samples were processed in an anaerobic chamber (Whitley DG250, Don Whitley Scientific, UK; 85% N_2_, 10% H_2_, 5% CO_2_) to minimize oxygen exposure. Aliquots intended for transplantation or sequencing were flash-frozen in liquid nitrogen and stored at −80°C to preserve microbial viability and community integrity (28).

### 16S rRNA sequencing

Fecal genomic DNA was extracted using the HiPure Stool DNA Mini Kit (Magen, Guangzhou, China) following the manufacturer’s protocol. The V3-V4 hypervariable regions of bacterial 16S rRNA genes were amplified with specific primers (341F: 5’-CCTACGGGNGGCWGCAG-3’; 805R: 5’-GACTACHVGGGTATCTAATCC-3’) in 30 μL reaction volumes. Thermal cycling parameters included: initial denaturation at 95°C for 3 min; 25 cycles of denaturation (95°C, 30 s), annealing (55°C, 30 s), and extension (72°C, 15 s); followed by final extension at 72°C for 5 min.

PCR products were purified using the Agencourt AMPure XP system (Beckman Coulter, USA) and quality-verified through 1.5% agarose gel electrophoresis. Target fragments were recovered using the AxyPrep DNA Gel Extraction Kit (AP-GX-250G, Corning, USA). Library concentrations were quantified with the Qubit dsDNA HS Assay Kit (Thermo Fisher Scientific, USA) and sequenced on the Illumina MiSeq platform (2×300 bp paired-end) for subsequent bioinformatic analysis.

### Bioinformatic analysis

The minimum read depth for each sample was 50,000 reads and the Q30 value was >80%. Following initial quality assessment with FastQC, raw sequencing reads were processed through the DADA2 pipeline implementing the following parameters: truncation of reads containing ambiguous bases (N) or exceeding estimated error thresholds (>2), succeeded by *de novo* chimera elimination. High-fidelity sequences were clustered into operational taxonomic units (OTU) at 97% similarity using the UPARSE algorithm. Taxonomic classification was executed against the SILVA 138.1 reference database. α-diversity was quantified using Shannon indices, whereas beta diversity analysis was conducted through the application of Bray-Curtis dissimilarity matrices and Bray-Curtis, with results visualized via principal coordinates analysis (PCoA). Differential taxa identification was conducted via LEfSe with significance thresholds set at LDA score >3.0 and *p* < 0.05. All statistical analyses and visualizations were implemented in R statistical environment (v4.4.2).

### Donor screening

LEfSe was employed to identify discriminative microbial taxa between IBD patients and healthy controls. Health-enriched taxa (LDA score >2.0, *p* < 0.05) were prioritized for donor stratification. Healthy donors were ranked according to median relative abundance thresholds of these signature taxa, with the top and bottom quartiles designated as high-abundance (H donor) and low-fabundance donor(L donor) cohorts, respectively.

### Sample preparation

Fecal specimens (50 g) from healthy donors were transferred into an anaerobic chamber and homogenized in sterile, pre-reduced physiological saline (0.9% NaCl) at a 1:3 (w/v) ratio using homogenizers. The resultant slurry was subsequently filtered through sterile nylon mesh under anaerobic conditions to yield bacterial suspensions. These suspensions were then aliquoted into sterile, anaerobic cryovials, rapidly frozen in liquid nitrogen, and cryopreserved at -80°C (28, 29).

### Animals and experimental design

Male C57BL/6J mice (6–8 weeks old) were used in this study to ensure consistency and avoid potential confounding effects of hormonal fluctuations that may influence the gut microbiota and immune responses. 66 male C57BL/6J mice were purchased from the germfree animal platform of Huazhong Agricultural University. Mice were maintained under controlled SPF conditions with temperature 25 ± 2°C, relative humidity 45-60%, and 12 h light/dark cycles. After 7-day acclimatization with ad libitum access to food and water, animals were randomly allocated into 11 experimental groups: control group, Model group (DSS), high-abundance donor A group (H-A), high-abundance donor B group (H-B), high-abundance donor C group (H-C), high-abundance donor D group (H-D), low-abundance donor A group (L-A), low-abundance donor B group (L-B), low-abundance donor C group (L-C), low-abundance donor D group (L-D), and the 5-ASA group. This study was approved by the Ethics Committee of Shenzhen Guangming District People’s Hospital (approval number: LL-KT-2024108).

Mice in all groups except the control group drank 3% DSS (MP Biomedicals, Santa Ana, United States) solution, and after 10 days, mice in the 5-ASA group (100 mg/kg, JiaxingSiCheng Chemical Co., Ltd., China) were administered by gavage daily. The other groups were given PBS, H and L donor fecal bacteria (0.2 mL) by gavage on days 5, 7, 9, 11, 13 and 15, respectively. Prior to administration, the fecal bacteria were thawed at room temperature for 30 minutes under anaerobic conditions and were immediately gavaged into germ-free mice within a biosafety cabinet that was continuously flushed with an anaerobic gas mixture to mitigate the risk of oxidation. During the experimental period, mice were scored with DAI, blood was taken from the orbits on day 19, the colon was taken to measure the length and photographed, and the contents of the mouse cecum were collected.

### DAI score

Disease Activity Index (DAI) scores, which assess body weight loss, stool viscosity, and the presence of blood in stool, were evaluated on days 1, 4, 7, 10, 13, 16, and 19. The scoring criteria are detailed **Table 1**.

**Table 1:** DAI score.

Grade	Weight loss (%)	Stool viscosity	Bloody stool acore
0	0	Normal	Normal colored stool
1	1-5%	Loose stool	Brown stool
2	5-10%	Loose stool	Reddish stool
3	10-20%	Diarrhea	Bloody stool
4	>20%	Diarrhea	Gross bleeding

### 16S rRNA sequencing and analysis of gut microbiota in IBD mice

Cecal contents were collected, immediately flash-frozen in liquid nitrogen, and stored at −80°C until further processing. Gut microbiota profiling and subsequent bioinformatic analysis were performed according to the protocols detailed in Methods 2.2 (16S rRNA sequencing) and 2.3 (Bioinformatic analysis).

### Statistical analysis

Statistical analyses were conducted in QIIME2 (v2024.2). α-Diversity was quantified using the Shannon index, with group differences assessed by Kruskal-Wallis rank sum test. Bray-Curtis distances were computed for the Anosim analysis, while Bray-Curtis distances were utilized for principal coordinate analysis (PCoA) to evaluate beta diversity. The significance of the findings was assessed through the application of PerMANOVA. LEfSe analysis was performed using Wilcoxon and Kruskal-Wallis tests for biomarker identification and Benjamini-Hochberg for multiple testing. Wilcoxon rank sum test was used to compare the groups in animal experiments. Data are presented as mean ± standard deviation, with *p* < 0.05 considered statistically significant.

## Result

This study enrolled 39 IBD patients and 42 HC. All participants were screened to exclude comorbidities. Demographic and clinical characteristics are summarized in **Table 2**.

**Table 2:** Demographic and clinical characteristics.

Group	IBD (n = 39)	HC (n =42)
Male/Female	25/14	19/23
Mean/median age (years)	43.2/42	43.7/41
Mean/median weight (kg)	62.7/65	62.4/66
Mean/median BMI (kg/m^2^)	21.8/23.8	21.7/22.8
Loose stools/diarrhea	24 (61.5%)/12 (30.8%)	0
Hematochezia	3 (7.7%)	0

### Dysbiosis of gut microbial community in IBD patients versus healthy controls

**Figure 1:**
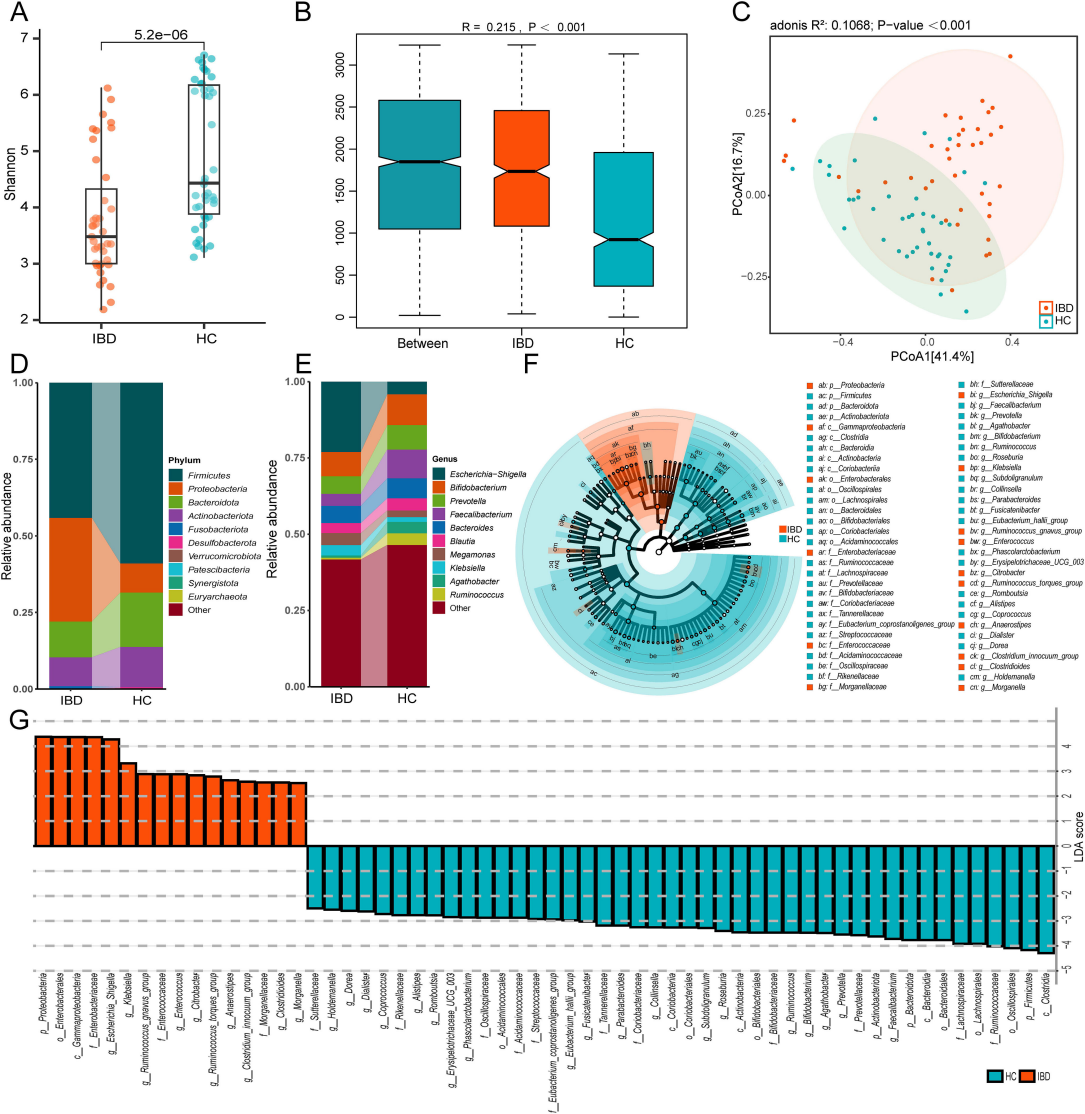
Distinct gut microbiota profiles between IBD patients and healthy controls (HC). **(A)** The Shannon index of gut microbiota with IBD compared to HC. **(B)** Anosim analysis based on Bray-Curtis distance. **(C)** Bray-Curtis distance-based PCoA. **(D)** Phylum-level taxonomic composition. **(E)** Genus-level taxonomic composition. **(F)** The cladogram shows differences in enriched taxa in IBD patients (red) compared to enriched taxa in controls (blue). **(G)** LDA effect size distribution (LDA Score>2). IBD and HC group (2 groups).

To investigate gut microbiota disparities between IBD patients and HC, we enrolled 39 treatment-naïve IBD patients and 42 age-matched HC. Fecal samples underwent 16S rRNA gene sequencing using the Illumina NovaSeq platform. α-diversity analysis revealed significantly lower Shannon indices in IBD patients compared to HC (*p* < 0.001) (**Figure 1A**), indicating reduced microbial diversity. β-diversity analysis is employed to investigate alterations in the overall composition of the gut microbiota. An Anosim analysis was conducted to assess whether the differences between groups were significantly greater than the differences observed within groups. The results demonstrated significant disparities between the IBD group and HC (R = 0.215, *p* < 0.001, **Figure 1B**). PCoA based on Bray-Curtis distances showed clear separation along the first principal coordinate (PCoA1: 41.3% variance explained) (**Figure 1C**), confirming structural divergence of microbial communities.

We conducted taxonomic profiling of the top 10 microbial species in IBD patients. At the phylum level, significant intergroup disparities were observed in Firmicutes, Proteobacteria, Bacteroidota, and Actinobacteriota, with IBD patients exhibiting marked enrichment of Proteobacteria (IBD: 33.8%, HC: 9.5%) (**Figure 1D**). Genus-level analysis revealed differential abundance of *Escherichia-Shigella*, *Bifidobacterium*, *Prevotella*, *Faecalibacterium*, *Bacteroides*, *Blautia*, *Megamonas*, *Klebsiella*, *Agathobacter*, and *Ruminococcus*. Notably, the gut microbiota of IBD patients exhibited marked enrichment of *Escherichia-Shigella* (5.5-fold increase), *Megamonas* (1.8-fold), and *Klebsiella* (2.2-fold) compared to healthy controls (**Figure 1E**). These genera (particularly *Escherichia-Shigella* and *Klebsiella*) are established pro-inflammatory pathogens implicated in mucosal inflammation (30, 31). Conversely, putative beneficial taxa including *Bifidobacterium*, *Faecalibacterium*, and *Agathobacter* showed 12%-53% reductions in relative abundance compared to controls (**Figure 1E**) (32, 33). Collectively, these findings substantiate IBD-associated dysbiosis characterized by depleted microbial diversity and taxonomic restructuring.

### Distinct compositional profiles of gut microbiota among different donor cohorts

**Figure 2:**
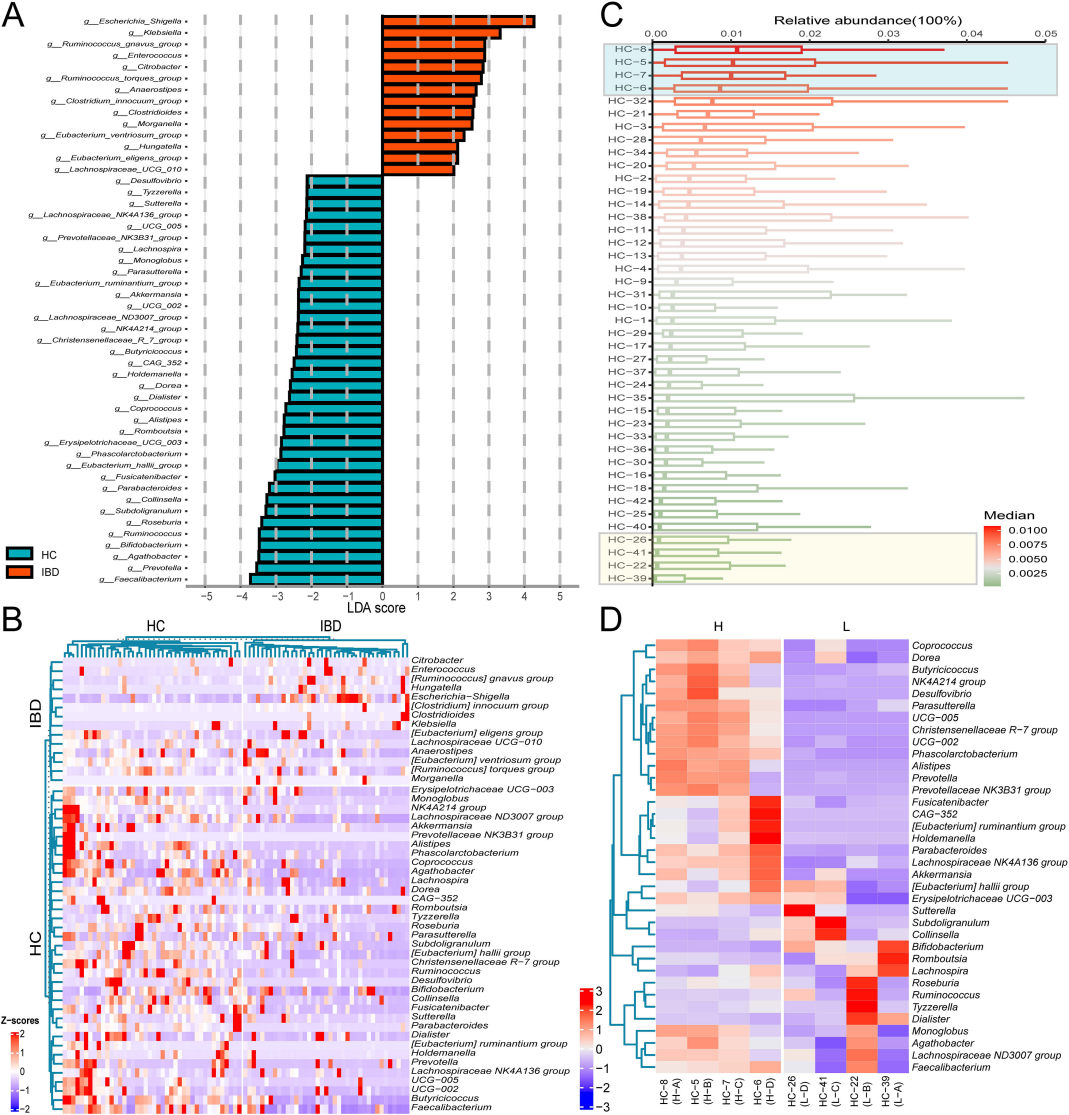
Differential microbial taxa across donor groups. **(A)** LDA effect size distribution of discriminant genera between IBD and HC groups. **(B)** Average relative abundance clustering heatmap of differential genera in 39 IBD patients versus 42 healthy controls. **(C)** Median abundance ranking of 36 putative beneficial genera in 42 healthy donor gut microbiota. **(D)** Differential genera average relative abundance clustering heatmap of 36 putative beneficial taxa across 8 donor gut microbiota. The labels “IBD” and “HC” on the left panel of **(B)** denote differentially enriched bacterial genera specific to the IBD group and HC group, respectively.

To characterize the donor-specific microbial profiles, we employed LEfSe to identify differentially abundant taxa between IBD patients and HC. LEfSe was employed to identify microbial biomarkers between groups. Phylogenetic cladograms generated through LDA scores (>2.0) and LEfSe analysis revealed taxonomic cladogram differences across microbial communities (**Figures 1F, G**). We further identified 50 bacterial genera with significant differences in abundance between IBD patients and HCs based on Lefse analysis (**Figure 2A**). Heatmap visualization revealed distinct enrichment patterns: 36 putative beneficial taxa (e.g., *Firmicutes*, *Bacteroidota*, *Actinobacteriota*, *Clostridia*) dominated HC donors, whereas 14 potentially pathogenic lineages (*Proteobacteria*, *Gammaproteobacteria*, *Enterobacterales*) were enriched in IBD donors (**Figure 2A**). Inter-donor heterogeneity in microbial stratification was evident (**Figure 2B**). Based on median relative abundance of the 36 beneficial genera, donors were stratified into 4 high-abundance (HC-8, HC-5, HC-7, HC-6) and 4 low-abundance (HC-26, HC-41, HC-22, HC-39) cohorts (**Figure 2C**). Comparative analysis demonstrated marked enrichment of *Coprococcus*, *Dorea*, and *Butyricicoccus* in H donors, with significantly reduced beneficial taxa in L donors (**Figure 2D**). Our findings demonstrate compositional stratification of gut microbiota across donor populations based on the relative abundance of health-associated bacterial genera.

### Fecal microbiota transplantation improves colitis in mice

**Figure 3:**
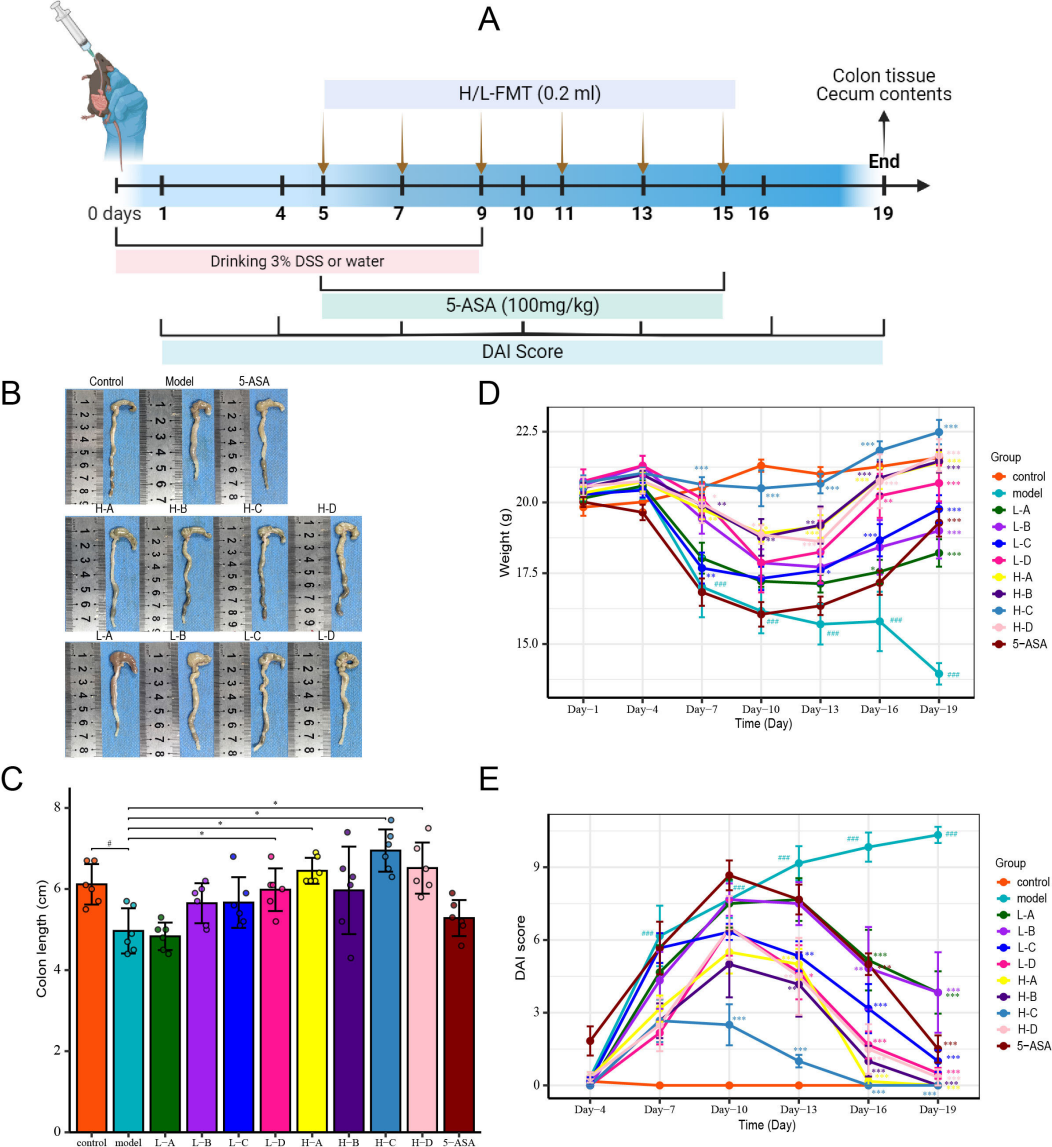
FMT alleviates DSS-induced murine IBD symptoms. **(A)** Schematic of DSS-induced IBD modeling. **(B)** Representative colon images showing gross morphology. **(C)** Colon length comparison across groups. **(D)** Body weight variation. **(E)** Disease activity index (DAI) scores. Data presented as mean ± SD (n=6). Compared with the control group: ^#^ *p*<0.05, ^###^ *p*<0.001; compared with the model group: *p<0.05, **p<0.01, ***p<0.001.

To investigate the therapeutic implications of donor microbial composition, we established a dextran sulfate sodium (DSS)-induced murine colitis model (**Figure 3A**). Mice were randomized to receive FMT from either H or L donors, alongside a control treatment of 5-ASA. DSS administration induced significant colonic mucosal damage, as evidenced by shortened colon length (**Figures 3B, C**). Disease progression was characterized by progressive weight loss, hematochezia, and diarrhea (**Figures 3D, E**). Notably, recipients of FMT exhibited significant attenuation of colonic injury, reversal of weight loss trajectories, and amelioration of hematochezia and diarrhea. Importantly, recipients of H-donor FMT demonstrated superior therapeutic efficacy compared to those receiving L-donor FMT and the 5-ASA treatment group. The results showed that the mice receiving FMT from H donors showed a significantly different trend in weight change from IBD mice on the 7th day, and IBD symptoms such as hematochezia and diarrhea were significantly improved on the 13th day. In contrast, mice receiving FMT from L donors showed significant disease improvement only after multiple treatments (**Figures 3B–E**). These findings collectively demonstrate that donor microbial composition critically determines FMT efficacy in ameliorating IBD pathology.

### Fecal microbiota transplantation reshapes gut microbial community structure in murine colitis

**Figure 4:**
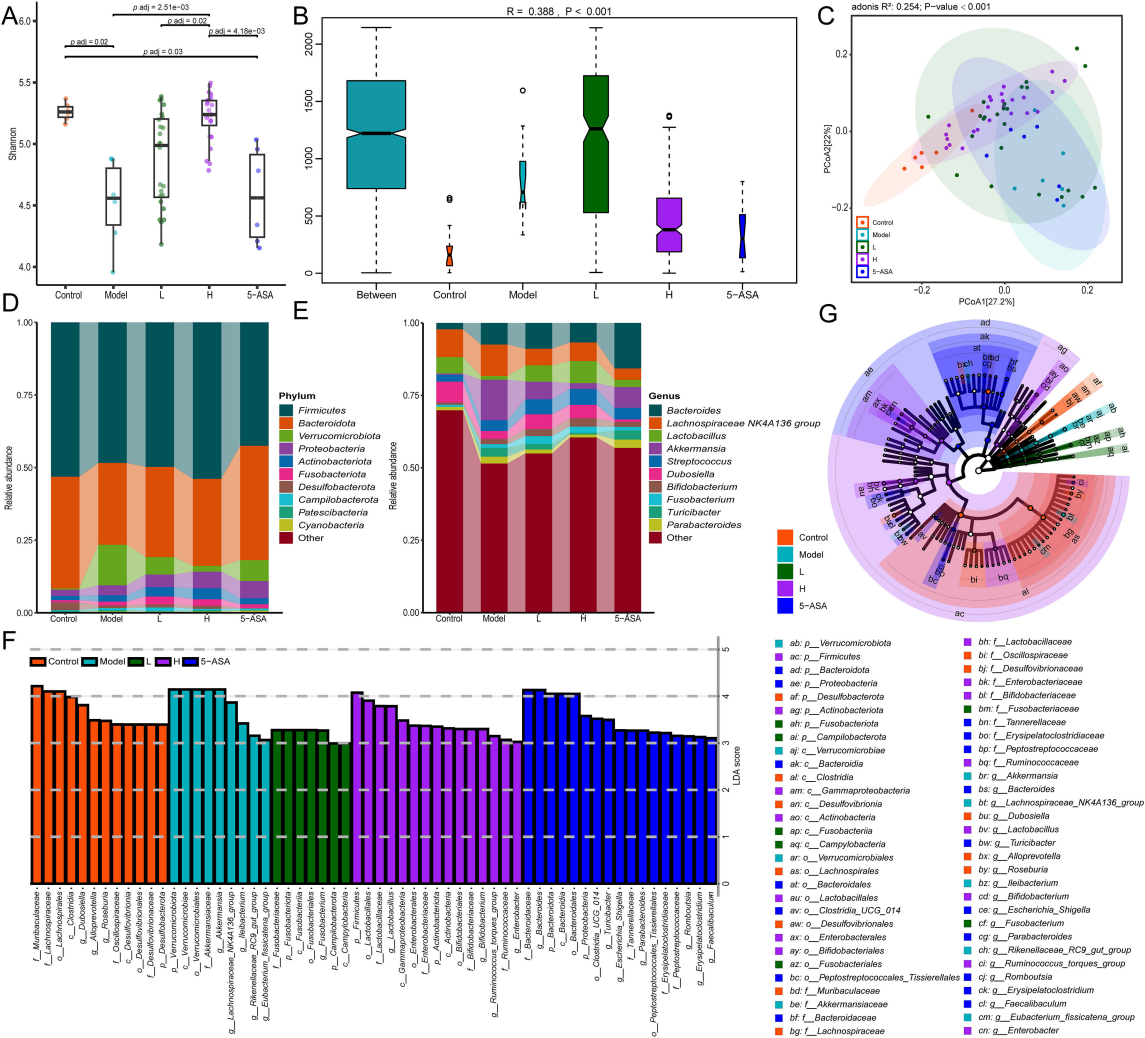
FMT ameliorates DSS-induced gut microbiota dysbiosis in murine models. **(A)** Shannon index of α-diversity across groups. **(B)** Anosim analysis based on Bray-Curtis distance. **(C)** Bray-Curtis distance-based PCoA. **(D)** Phylum-level taxonomic composition. **(E)** Genus-level taxonomic composition. **(G)** The cladogram illustrates the variations in enriched taxa across differentially enriched taxa for the control (red), model (cyan), H-group (green), L-group (purple), and 5-ASA (blue) groups. **(F)** LDA effect size distribution (LDA Score>3). Control, model, H, L, and 5-ASA group (5 groups).

To assess the impacts of donor microbial composition on microbial ecology, we conducted gut microbiota profiling in IBD mice following FMT. The α-diversity analysis revealed significantly reduced Shannon indices in IBD mice compared to control mice (*p* = 0.02), indicating dysbiosis (**Figure 4A**). Notably, recipients of H-donor FMT exhibited a marked restoration of α-diversity, approximating healthy controls (p < 0.001 vs model group), whereas L-donor recipients showed intermediate recovery (*p* = 0.02 vs H-group) (**Figure 4A**, **Supplementary Figure 1A**). Anosim analysis revealed statistically significant differences in gut microbiota across all groups ((R = 0.388, *p* < 0.001), (**Figure 4B**, **Supplementary Figure 1B**). The analysis of β-diversity employing Bray-Curtis-based PCoA revealed distinct microbial community restoration patterns (R (2) = 0.254, *p* = 0.001), the H group exhibited near-complete restitution of gut microbiota diversity, whereas the L group exhibited significantly lower diversity indices (**Figure 4C**).

Phylum-level analysis revealed significant microbial shifts in IBD mice, characterized by a decrease in the abundance of Firmicutes and Bacteroidota, accompanied by an increase in Verrucomicrobiota, Proteobacteria, Actinobacteriota, and Fusobacteriota (**Figure 4D**, **Supplementary Figure 1C**). The FMT intervention, particularly from H-donors, restored Firmicutes to near-normal levels, demonstrating superior efficacy compared to 5-aminosalicylic acid (5-ASA) treatment (**Figure 4D**). At the genus level, DSS-induced colitis mice exhibited marked microbial dysbiosis, characterized by an increased relative abundance of *Bacteroides* (3.4-fold), *Lachnospiraceae_NK4A136_group* (1.1-fold), *Akkermansia* (33.8-fold), *Streptococcus* (1.5-fold), *Bifidobacterium* (2.1-fold), *Fusobacterium* (1.9-fold), and *Parabacteroides* (2.0-fold), concomitant with significant depletion of beneficial taxa including *Lactobacillus* (0.23-fold) and *Dubosiella* (0.38-fold). Both FMT and 5-ASA treatments improved gut microbiota dysbiosis in IBD mice. Compared to untreated IBD mice, FMT intervention significantly enriched *Lactobacillus* (5.9-fold increase) and *Dubosiella* (1.6-fold increase)—the latter being a murine commensal known for its anti-inflammatory properties (34). Furthermore, FMT normalized the relative abundance of *Lachnospiraceae_NK4A136_group*, *Akkermansia*, *Turicibacter*, and *Parabacteroides* to near-physiological levels. Notably, H-donor FMT effectively normalized the DSS-induced elevation of *Bacteroides* (**Figure 4E**, **Supplementary Figure 1D**). These findings highlight the superior therapeutic efficacy of FMT compared to 5-ASA in microbial restoration, with H-donors achieving optimal ecological recovery.

### Donor-specific FMT restores gut microbial balance in mice with colitis

**Figure 5:**
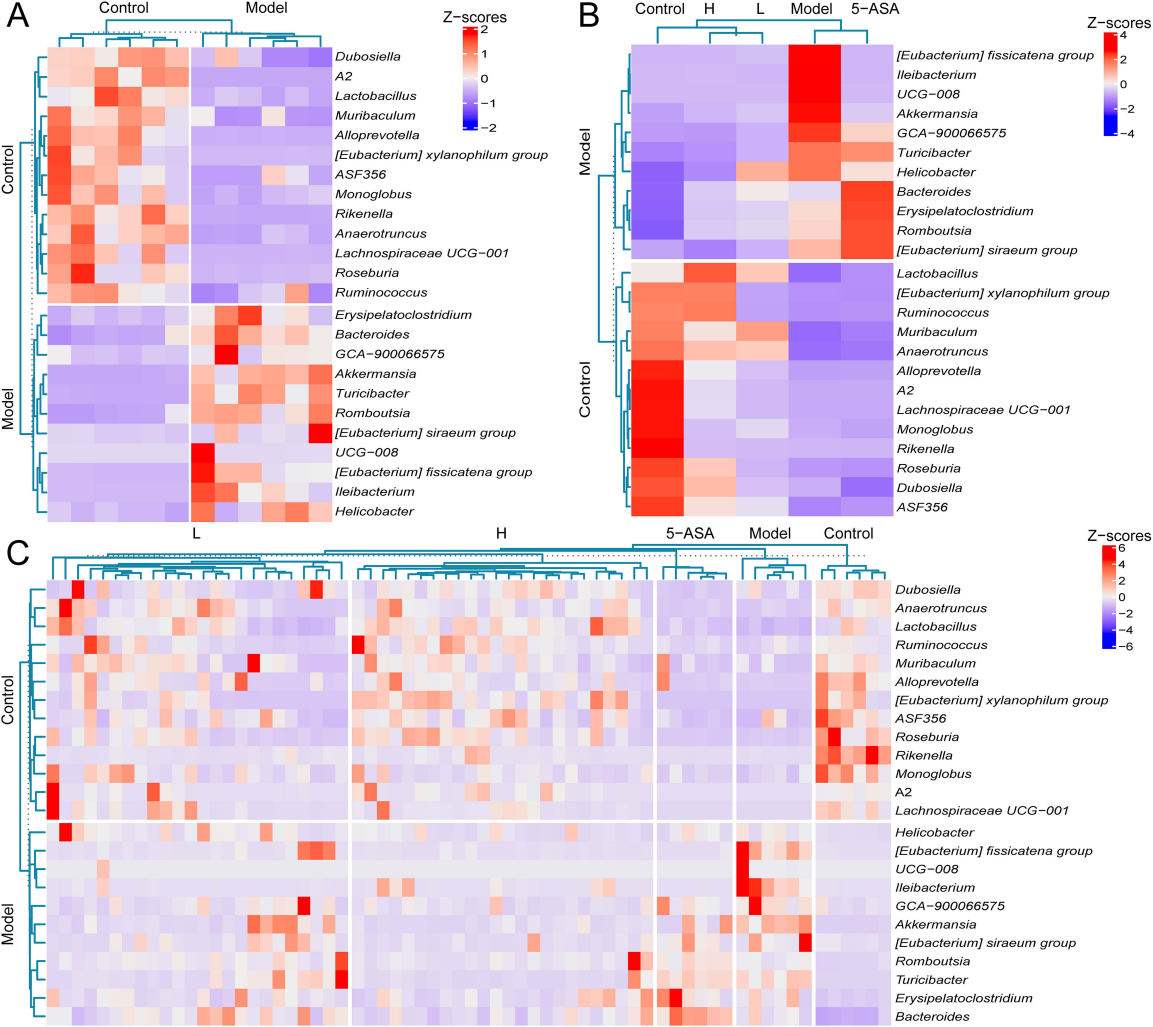
High-abundance donor FMT induces phylogenetic convergence toward control microbiota. **(A)** Average relative abundance clustering heatmap of discriminant microbial between control and model groups. **(B)** Average relative abundance clusteringheatmap of 24 differential genera across all experimental cohorts. **(C)** Taxon average relative abundance clustering heatmap of differential microbiota in 66 murine samples. The labels “control” and “model” on the left panel of **(A–C)** denote differentially enriched bacterial genera specific to the control group and model group, respectively.

Subsequently, we performed LEfSe analysis on the gut microbiota of IBD mice and treatment groups. The LDA scores (>3.0) revealed taxonomically enriched biomarkers among the cohorts, indicating a compositional divergence in the microbiota (**Figure 4F**). Phylogenetic cladograms illustrated structural community disparities (**Figure 4G**). Significant differences in microorganisms between control and IBD mice were assessed using LEfSe. At the genus level, a total of 24 distinct genera, including *Akkermansia*, *Bacteroides*, *Lactobacillus*, and *Dubosiella*, were identified as enriched in the two groups. IBD mice exhibited an enrichment of *Helicobacter*, *Akkermansia*, and 11 other genera, while control mice were enriched with a total of 13 genera, including *Roseburia* and *Dubosiella* (**Figure 5A**). We conducted a comparative analysis of 24 discriminant genera across treatment cohorts. Heatmap analysis demonstrated that 5-ASA treatment significantly reduced the abundance of *[Eubacterium]_fissicatena_group*, *Ileibacterium*, *UCG-008*, and *Akkermansia* compared to IBD controls. While L-donor FMT attenuated potentially detrimental taxa, only *Lactobacillus*, *Muribaculum*, and *Anaerotruncus* approached control levels. In contrast, H-donor FMT not only reduced detrimental taxa but also enhanced beneficial genera, including *Lactobacillus*, *[Eubacterium]_xylanophilum_group*, *Anaerotruncus*, *Romboutsi*, and *Dubosiella*, to near-physiological ranges (**Figure 5B**). Individual recipient analysis through differential taxa average relative abundance clustering heatmaps demonstrated significantly higher therapeutic response rates compared to both L- donor FMT and 5-ASA interventions, along with enhanced microbial restoration in H-donor FMT cohorts (**Figure 5C**). These findings establish the superior capacity of H-donor FMT to ameliorate IBD-associated dysbiosis.

## Discussion

IBD has emerged as a significant global health challenge, with incidence rates escalating disproportionately in rapidly urbanizing regions (1, 35). Current therapeutic regimens remain suboptimal, constrained by adverse effects and high relapse rates that hinder sustained remission (36). This situation underscores the urgent need to develop novel therapeutic strategies targeting the pathophysiology of IBD. Mounting evidence implicates gut microbiota dysbiosis as a key driver of IBD progression. Consequently, FMT has gained prominence as a microbiota-targeted intervention (37, 38). though clinical efficacy demonstrates marked inter-donor variability (25). A randomized trial involving 38 participants demonstrated remission rates of 39% with donor B-derived FMT compared to only 10% with other donors (39). Similarly, only 2 of 8 patients achieved endoscopic and clinical remission following donor-stratified FMT (40). This evidence suggests that the efficacy of FMT in treating IBD is donor-dependent. Our study aims to explore how the compositional features of donor microbiota, inferred from 16S rRNA profiles, influence the outcomes of fecal microbiota transplantation in a murine colitis model. This approach offers a compositional framework for understanding donor variability in preclinical FMT responses.

We conducted comparative analysis of gut microbiota diversity in fecal samples from 39 IBD patients and 42 healthy donors. Firmicutes, Proteobacteria, Bacteroidota, and Actinobacteriota are predominant in healthy gut microbiota (15). Our findings reaffirm this observation; however, an abnormal increase in Proteobacteria was noted in patients with IBD. Notably, the inflammatory symptoms observed in IBD patients may be associated with an increase in pro-inflammatory genera such as *Escherichia-Shigella*, *Megamonas*, and *Klebsiella*, alongside a decrease in anti-inflammatory genera including *Bifidobacterium*, *Faecalibacterium*, and *Agathobacter*. The abundance and compositional of donor microbiota may be crucial for enhancing the efficacy of FMT in the treatment of IBD (40). We identified eight healthy donors with distinct microbial compositional profiles in their gut microbiota by screening for potentially beneficial microbiota enriched in healthy individuals and ranking them according to their median abundance. The top four donors were classified as having high abundance microbiota donor, while the bottom four were categorized as having low abundance microbiota donor.

In the IBD mouse model, we demonstrated that FMT effectively alleviated symptoms of IBD, with H donor FMT exhibiting superior therapeutic effects. Additionally, we conducted a detailed analysis of changes in the gut microbiota of the mice using 16S rRNA sequencing. DSS-induced alterations in gut microbiota diversity and species distribution were observed in mice (41). In contrast, H donor FMT alleviated DSS-induced IBD symptoms by ameliorating microbial dysbiosis and restoring gut microbiota homeostasis. DSS-induced IBD mice exhibited marked enrichment of *Bacteroides*, *Streptococcus*, *Fusobacterium*, *Akkermansia*, and *Parabacteroides* concomitant with significant depletion of beneficial taxa including *Lactobacillus*, *Dubosiella (* 34). *Bacteroides* is a key commensal genus within the gut microbiota; however, its dysregulated expansion is associated with various disease states (42). *Streptococcus* and *Fusobacterium* are recognized as pathogenic genera (43, 44). The functional roles of *Parabacteroides* and *Akkermansia* remain debated. While *Parabacteroides* is generally associated with anti-inflammatory properties, its paradoxical enrichment in DSS-induced murine colitis models suggests context-dependent pro-inflammatory potential (45). *Akkermansia* demonstrates dual functionality: mucosal barrier restoration attenuating colonic inflammation versus colitis exacerbation under microenvironmental dysbiosis (46, 47). *Dubosiella* a genus known for its anti-inflammatory properties, experimental studies have shown that *Dubosiella* maintains a balanced immune response in the intestine by reducing the production of pro-inflammatory cytokines and promoting a favorable gut microbiome composition (34, 48). Similarly, *Lactobacillus*, a well-known probiotic genus, has been shown to enhance regulatory T cell (Treg) activity and inhibit excessive inflammation, and supplementation with *Lactobacillus* can improve clinical outcomes in patients with IBD by restoring microbial diversity and promoting immune tolerance (49, 50). H donor FMT induced gut microbiota remodeling through enrichment of beneficial taxa (*Lactobacillus*, *Dubosiella*) and suppression of potentially detrimental taxa (*Bacteroides*, *Streptococcus*, *Turicibacter*). Microbial profiles in the H-group demonstrated phylogenetic convergence with controls. These findings establish donor microbiota composition as a critical determinant of FMT efficacy in IBD management.

Our findings indicate that the therapeutic efficacy of FMT in IBD correlates with the functional characteristics of donor microbiota. We observed that transplantation of high-abundance donor fecal microbiota demonstrated the highest success rate in IBD treatment. This phenomenon may be associated with the elevated abundance of beneficial bacterial genera in high-abundance microbiota, including *Coprococcus*, *Dorea*, and *Butyricicoccus*.e.g. Specifically, *Coprococcus* and *Butyricicoccus*, as effective probiotics, alleviate IBD symptoms by reducing pro-inflammatory cytokine concentrations, *Butyricicoccus pullicaecorum*, a butyrate-producing bacterium, can lower myeloperoxidase (MPO) levels and mitigate the inflammatory response associated with IBD, in addition, studies have demonstrated that supplementation with *Coprococcus eutactus* can effectively improve IBD, alleviate weight loss, and reduce the concentration of pro-inflammatory cytokines in the body, while Dorea contributes to maintaining intestinal mucosal barrier integrity through the production of SCFAs (51–53).

Recent studies have highlighted the concept of ‘super-donors’, individuals with exceptional microbial diversity and functional richness, who have shown improved outcomes in fecal microbiota transplantation (FMT) for IBD patients (54). Super-donors are thought to contribute to therapeutic success due to their ability to restore microbial balance and function in the recipient’s gut microbiota. These donors are characterized by a balanced microbial community composition, which promotes homeostasis and potentially suppresses inflammation through beneficial microbiota-host interactions (55). In particular, super donor have been shown to modulate host immune responses, leading to better clinical outcomes (56, 57). Microbiota-host interactions play a pivotal role in FMT efficacy. These interactions include the modulation of immune system activity through the production of short-chain fatty acids (SCFAs), which influence intestinal permeability and immune cell regulation (58). Furthermore, specific microbial metabolites can either promote or inhibit inflammatory processes, highlighting the importance of microbial ecology and metabolite dynamics in IBD treatment (59). Current donor screening protocols prioritize individuals without infections or chronic diseases, yet factors such as age, sex, and dietary habits significantly influence gut microbiota composition (60, 61). Implementing donor-recipient matching based on microbial enterotypes may optimize FMT outcomes. For instance, the enterotype-based donor selection (EDS) model employs personalized treatment regimens by matching donors and recipients across complementary enterotypes, thereby introducing synergistic metabolic functions to improve symptom resolution (62, 63). Although our study did not incorporate enterotype-based matching, experimental data revealed that mice receiving high-abundance donor microbiota exhibited superior gut microbiota restoration and therapeutic response rates.

Our findings demonstrate an association between gut microbiota composition and therapeutic outcomes in IBD, identified potential beneficial bacterial taxa, several methodological limitations should be acknowledged. First, this study relied solely on 16S rRNA sequencing, which, while effective for broad taxonomic profiling, lacks species-level resolution and cannot directly assess microbial function. Future work should incorporate metagenomics or metabolomics for deeper functional insights. Second, while the DSS-induced colitis mouse model allowed us to evaluate donor-dependent effects in a controlled environment, it does not replicate the full immunological and microbial complexity of human IBD. Therefore, our findings should be considered preliminary and hypothesis-generating. Validation in humanized microbiota mouse models and well-characterized clinical cohorts will be needed to confirm translational relevance. Third, dietary intake was not controlled or recorded. Participants maintained their usual diets, which may have introduced variability in microbiota composition. While this reflects real-world conditions, it also poses a potential confounding factor. Future studies should consider dietary assessment or standardization to reduce this bias. Fourth, our sample size, while sufficient to reveal consistent trends, was limited, and future studies should increase both the number of human donors and recipient animals to enhance statistical power and generalizability. Finally, donor classification was based on relative abundance of health-associated genera without functional validation, which could be further refined by integrating microbial activity profiling in follow-up studies.

## Conclusions

This investigation systematically evaluated the impact of donor microbiota composition on FMT efficacy in IBD. Comparative analysis of gut microbiota profiles in 39 IBD patients and 42 healthy controls revealed diminished microbial diversity and aberrant enrichment of specific genera in IBD cohorts. Through targeted screening of healthy donors based on beneficial microbiota abundance, we established high-abundance and low-abundance donor cohorts. Employing a DSS-induced murine colitis model, FMT intervention demonstrated significant symptom alleviation and microbial dysbiosis amelioration. Notably, H donor FMT exhibited superior capacity to restore microbial diversity and enhance therapeutic outcomes. These findings suggest that donor microbial composition and taxonomic structure are key contributors to FMT efficacy, informing future development of personalized microbiota-based therapies for IBD.

## Data Availability

Raw 16S rRNA sequencing data have been deposited in the National Center for Biotechnology Information (NCBI) with study, accession no. PRJNA1240002.

## Supplementary material

Supplementary Figure 1 High-function donor FMT exhibits superior efficacy in ameliorating IBD-associated dysbiosis compared to other therapeutic cohorts. **(A)** Shannon index measuring α-diversity among IBD and treatment groups. **(B)** Anosim analysis based on Bray-Curtis distance. **(C)** Phylum-level taxonomic composition. **(D)** Genus-level taxonomic composition. Experimental cohorts: Control, model, H-A, H-B, H-C, H-D, L-A, L-B, L-C, L-D, and 5-ASA group (11 groups).

## References

[1] Ng SC Shi HY Hamidi N Underwood FE Tang W Benchimol EI. Worldwide incidence and prevalence of inflammatory bowel disease in the 21st century: a systematic review of population-based studies. Lancet. (2017) 390:2769–78. doi: 10.1016/S0140-6736(17)32448-0, PMID: 29050646

[2] Kuenzig ME Fung SG Marderfeld L Mak JWY Kaplan GG Ng SC. Twenty-first century trends in the global epidemiology of pediatric-onset inflammatory bowel disease: systematic review. Gastroenterology. (2022) 162:1147–1159.e4. doi: 10.1053/j.gastro.2021.12.282, PMID: 34995526

[3] Li C-J Wang Y-K Zhang S-M Ren M-D He S-X. Global burden of inflammatory bowel disease 1990-2019: A systematic examination of the disease burden and twenty-year forecast. World J Gastroenterol. (2023) 29:5751–67. doi: 10.3748/wjg.v29.i42.5751, PMID: 38075848 PMC10701338

[4] Cai Z Wang S Li J. Treatment of inflammatory bowel disease: A comprehensive review. Front Med. (2021) 8:765474. doi: 10.3389/fmed.2021.765474, PMID: 34988090 PMC8720971

[5] Di Rienzo A Marinelli L Dimmito MP Toto EC Di Stefano A Cacciatore I. Advancements in inflammatory bowel disease management: from traditional treatments to monoclonal antibodies and future drug delivery systems. Pharmaceutics. (2024) 16:1185. doi: 10.3390/pharmaceutics16091185, PMID: 39339221 PMC11435298

[6] Rutgeerts P Geboes K Vantrappen G Beyls J Kerremans R Hiele M. Predictability of the postoperative course of Crohn’s disease. Gastroenterology. (1990) 99(4):956–63. doi: 10.1016/0016-5085(90)90613-6, PMID: 2394349

[7] Bhakta A Tafen M Glotzer O Ata A Chismark AD Valerian BT. Increased incidence of surgical site infection in IBD patients. Dis Colon Rectum. (2016) 59:316. doi: 10.1097/DCR.0000000000000550, PMID: 26953990

[8] Garavaglia B Vallino L Amoruso A Pane M Ferraresi A Isidoro C. The role of gut microbiota, immune system, and autophagy in the pathogenesis of inflammatory bowel disease: Molecular mechanisms and therapeutic approaches. Asp Mol Med. (2024) 4:100056. doi: 10.1016/j.amolm.2024.100056

[9] Sugihara K Kamada N. Metabolic network of the gut microbiota in inflammatory bowel disease. Inflamm Regen. (2024) 44:11. doi: 10.1186/s41232-024-00321-w, PMID: 38443988 PMC10913301

[10] Ma ZF Lee YY. The role of the gut microbiota in health, diet, and disease with a focus on obesity. Foods. (2025) 14:492. doi: 10.3390/foods14030492, PMID: 39942085 PMC11817362

[11] Campbell C McKenney PT Konstantinovsky D Isaeva OI Schizas M Verter J. Bacterial metabolism of bile acids promotes generation of peripheral regulatory T cells. Nature. (2020) 581:475–9. doi: 10.1038/s41586-020-2193-0, PMID: 32461639 PMC7540721

[12] Macfarlane S Macfarlane GT. Regulation of short-chain fatty acid production. Proc Nutr Soc. (2003) 62:67–72. doi: 10.1079/PNS2002207, PMID: 12740060

[13] Zong X Fu J Xu B Wang Y Jin M. Interplay between gut microbiota and antimicrobial peptides. Anim Nutr. (2020) 6:389–96. doi: 10.1016/j.aninu.2020.09.002, PMID: 33364454 PMC7750803

[14] Ni J Wu GD Albenberg L Tomov VT. Gut microbiota and IBD: causation or correlation? Nat Rev Gastroenterol Hepatol. (2017) 14:573–84. doi: 10.1038/nrgastro.2017.88, PMID: 28743984 PMC5880536

[15] Lloyd-Price J Arze C Ananthakrishnan AN Schirmer M Avila-Pacheco J Poon TW. Multi-omics of the gut microbial ecosystem in inflammatory bowel diseases. Nature. (2019) 569:655–62. doi: 10.1038/s41586-019-1237-9, PMID: 31142855 PMC6650278

[16] Prosberg M Bendtsen F Vind I Petersen AM Gluud LL. The association between the gut microbiota and the inflammatory bowel disease activity: a systematic review and meta-analysis. Scand J Gastroenterol. (2016) 51:1407–15. doi: 10.1080/00365521.2016.1216587, PMID: 27687331

[17] Dupraz L Magniez A Rolhion N Richard ML Costa GD Touch S. Gut microbiota-derived short-chain fatty acids regulate IL-17 production by mouse and human intestinal γδ T cells. Cell Rep. (2021) 36(1):109332. doi: 10.1016/j.celrep.2021.109332, PMID: 34233192

[18] Macia. L Tan J Vieira AT Leach K Stanley D Luong S. Metabolite-sensing receptors GPR43 and GPR109A facilitate dietary fibre-induced gut homeostasis through regulation of the inflammasome. Nat Commun. (2015) 6:6734. doi: 10.1038/ncomms7734, PMID: 25828455

[19] Cai J Sun L Gonzalez FJ. Gut microbiota-derived bile acids in intestinal immunity, inflammation, and tumorigenesis. Cell Host Microbe. (2022) 30:289–300. doi: 10.1016/j.chom.2022.02.004, PMID: 35271802 PMC8923532

[20] Weingarden AR Vaughn BP. Intestinal microbiota, fecal microbiota transplantation, and inflammatory bowel disease. Gut Microbes. (2017) 8(3):238–52. doi: 10.1080/19490976.2017.1290757, PMID: 28609251 PMC5479396

[21] Quraishi MN Widlak M Bhala N Moore D Price M Sharma N. Systematic review with meta-analysis: the efficacy of faecal microbiota transplantation for the treatment of recurrent and refractory Clostridium difficile infection. Aliment Pharmacol Ther. (2017) 46(5):479–93. doi: 10.1111/apt.14201, PMID: 28707337

[22] Pu D Yao Y Zhou C Liu R Wang Z Liu Y. FMT rescues mice from DSS-induced colitis in a STING-dependent manner. Gut Microbes. (2024) 16(1):2397879. doi: 10.1080/19490976.2024.2397879, PMID: 39324491 PMC11441074

[23] Yang Y Zheng X Wang Y Tan X Zou H Feng S. Human fecal microbiota transplantation reduces the susceptibility to dextran sulfate sodium-induced germ-free mouse colitis. Front Immunol. (2022) 13:836542. doi: 10.3389/fimmu.2022.836542, PMID: 35237276 PMC8882623

[24] Ianiro G Bibbò S Porcari S Settanni CR Giambò F Curta AR. Fecal microbiota transplantation for recurrent C. difficile infection in patients with inflammatory bowel disease: experience of a large-volume European FMT center. Gut Microbes. (2021) 13(1):1994834. doi: 10.1080/19490976.2021.1994834, PMID: 34709989 PMC8555518

[25] Sokol H Landman C Seksik P Berard L Montil M Nion-Larmurier I. Fecal microbiota transplantation to maintain remission in Crohn’s disease: a pilot randomized controlled study. Microbiome. (2020) 8:1–14. doi: 10.1186/s40168-020-0792-5, PMID: 32014035 PMC6998149

[26] Thomas H. FMT induces clinical remission in ulcerative colitis. Nat Rev Gastroenterol Hepatol. (2017) 14:196–6. doi: 10.1038/nrgastro.2017.27, PMID: 28250470

[27] Porcari S Baunwall SMD Occhionero AS Ingrosso MR Ford AC Hvas CL. Fecal microbiota transplantation for recurrent C. difficile infection in patients with inflammatory bowel disease: A systematic review and meta-analysis. J Autoimmun. (2023) 141:103036. doi: 10.1016/j.jaut.2023.103036, PMID: 37098448

[28] Porcari S Baunwall SMD Occhionero AS Ingrosso MR Ford AC Hvas CL. Procedures for fecal microbiota transplantation in murine microbiome studies. Front Cell Infect Microbiol. (2021) 11:711055. doi: 10.3389/fcimb.2021.711055, PMID: 34621688 PMC8490673

[29] NHC Society of Parenteral and Enteral Nutrition Chinese Medical Association Intestinal Microecology Cooperative Group Chinese Society for Parenteral and Enteral Nutrition. Expert consensus on clinical application management of fecal microbiota transplantation (2022 edition). Chin J Gastrointest Surg. (2022) 25:747–56. doi: 10.3760/cma.j.cn441530-20220725-00324, PMID: 36117364

[30] Dong N Yang X Chan EW-C Zhang R Chen S. Klebsiella species: Taxonomy, hypervirulence and multidrug resistance. eBioMedicine. (2022) 79:103998. doi: 10.1016/j.ebiom.2022.103998, PMID: 35405387 PMC9010751

[31] Yang L Xiang Z Zou J Zhang Y Ni Y Yang J. Comprehensive analysis of the relationships between the gut microbiota and fecal metabolome in individuals with primary sjogren’s syndrome by 16S rRNA sequencing and LC-MS-based metabolomics. Front Immunol. (2022) 13. doi: 10.3389/fimmu.2022.874021, PMID: 35634334 PMC9130595

[32] Hertz S Anderson JM Nielsen HL Schachtschneider C McCauley KE Özçam M. Fecal microbiota is associated with extraintestinal manifestations in inflammatory bowel disease. Ann Med. (2024) 56(1):2338244. doi: 10.1080/07853890.2024.2338244, PMID: 38648495 PMC11036898

[33] Martin R Rios-Covian D Huillet E Auger S Khazaal S Bermúdez-Humarán LG. Faecalibacterium: a bacterial genus with promising human health applications. FEMS Microbiol Rev. (2023) 47(4):fuad039. doi: 10.1093/femsre/fuad039, PMID: 37451743 PMC10410495

[34] Zhang Y Tu S Ji X Wu J Meng J Gao J. Dubosiella newyorkensis modulates immune tolerance in colitis via the L-lysine-activated AhR-IDO1-Kyn pathway. Nat Commun. (2024) 15. doi: 10.1038/s41467-024-45636-x, PMID: 38351003 PMC10864277

[35] Kaplan GG Ng SC. Understanding and preventing the global increase of inflammatory bowel disease. Gastroenterology. (2017) 152:313–321.e2. doi: 10.1053/j.gastro.2016.10.020, PMID: 27793607

[36] Yeshi K Jamtsho T Wangchuk P. Current treatments, emerging therapeutics, and natural remedies for inflammatory bowel disease. Molecules. (2024) 29:3954. doi: 10.3390/molecules29163954, PMID: 39203033 PMC11357616

[37] Haneishi Y Furuya Y Hasegawa M Picarelli A Rossi M Miyamoto J. Inflammatory bowel diseases and gut microbiota. Int J Mol Sci. (2023) 24:3817. doi: 10.3390/ijms24043817, PMID: 36835245 PMC9958622

[38] Zuo T Ng SC. The gut microbiota in the pathogenesis and therapeutics of inflammatory bowel disease. Front Microbiol. (2018) 9:365492. doi: 10.3389/fmicb.2018.02247, PMID: 30319571 PMC6167487

[39] Moayyedi P Surette MG Kim PT Libertucci J Wolfe M Onischi C. Fecal microbiota transplantation induces remission in patients with active ulcerative colitis in a randomized controlled trial. Gastroenterology. (2015) 149:102–109.e6. doi: 10.1053/j.gastro.2015.04.001, PMID: 25857665

[40] Vermeire S Joossens M Verbeke K Wang J Machiels K Sabino J. Donor species richness determines faecal microbiota transplantation success in inflammatory bowel disease. J Crohns Colitis. (2015) 10:387. doi: 10.1093/ecco-jcc/jjv203, PMID: 26519463 PMC4946755

[41] Fan L Qi Y Qu S Chen X Li A Hendi M. B. adolescentis ameliorates chronic colitis by regulating Treg/Th2 response and gut microbiota remodeling. Gut Microbes. (2021) 13(1):1–17. doi: 10.1080/19490976.2020.1826746, PMID: 33557671 PMC7889144

[42] Wexler HM. Bacteroides: the good, the bad, and the nitty-gritty. Clin Microbiol Rev. (2007) 20(4):593–621. doi: 10.1128/cmr.00008-07, PMID: 17934076 PMC2176045

[43] Kostic AD Gevers D Pedamallu CS Michaud M Duke F Earl AM. Genomic analysis identifies association of Fusobacterium with colorectal carcinoma. Genome Res. (2012) 22(2):292–8. doi: 10.1101/gr.126573.111, PMID: 22009990 PMC3266036

[44] Claridge JE 3rd Attorri S Musher DM Hebert J Dunbar S. Streptococcus intermedius, Streptococcus constellatus, and Streptococcus anginosus (‘Streptococcus milleri group’) are of different clinical importance and are not equally associated with abscess. Clin Infect Dis. (2001) 32(10):1511–5. doi: 10.1086/320163, PMID: 11317256

[45] Ezeji JC Sarikonda DK Hopperton A Erkkila HL Cohen DE Martinez SP. Parabacteroides distasonis: intriguing aerotolerant gut anaerobe with emerging antimicrobial resistance and pathogenic and probiotic roles in human health. Gut Microbes. (2021) 13(1):1922241. doi: 10.1080/19490976.2021.1922241, PMID: 34196581 PMC8253142

[46] Seregin SS Golovchenko N Schaf B Chen J Pudlo NA Mitchell J. NLRP6 protects il10-/- mice from colitis by limiting colonization of akkermansia muciniphila. Cell Rep. (2017) 19(10):2174. doi: 10.1016/j.celrep.2017.05.074, PMID: 28591587

[47] Bian X Wu W Yang L Lv L Wang Q Li Y. Administration of akkermansia muciniphila ameliorates dextran sulfate sodium-induced ulcerative colitis in mice. Front Microbiol. (2019) 10(7):873. doi: 10.3389/fmicb.2019.02259, PMID: 31632373 PMC6779789

[48] Li. Y Wang W Liu Y Li S Wang J Hou L. Diminished immune response and elevated abundance in gut microbe dubosiella in mouse models of chronic colitis with GBP5 deficiency. Biomolecules. (2024) 14(7):873. doi: 10.3390/biom14070873, PMID: 39062588 PMC11274912

[49] Li C Peng K Xiao S Long Y Yu Q. The role of Lactobacillus in inflammatory bowel disease: from actualities to prospects. Cell Death Discov. (2023) 9(1):361. doi: 10.1038/s41420-023-01666-w, PMID: 37773196 PMC10541886

[50] Wang H Zhou C Huang J Kuai X Shao X. The potential therapeutic role of Lactobacillus reuteri for treatment of inflammatory bowel disease. Am J Transl Res. (2020) 12:1569. 32509162 PMC7270012

[51] Yang R Shan S Shi J Li H An N Li S. Coprococcus eutactus, a Potent Probiotic, Alleviates Colitis via Acetate-Mediated IgA Response and Microbiota Restoration. J Agric Food Chem. (2023) 71(7):3273–84. doi: 10.1021/acs.jafc.2c06697, PMID: 36786768

[52] Eeckhaut V Machiels K Perrier C Romero C Maes S Flahou B. Butyricicoccus pullicaecorum in inflammatory bowel disease. Gut. (2013) 62:1745–52. doi: 10.1136/gutjnl-2012-303611, PMID: 23263527

[53] Chen W Li Y Wang W Gao S Hu J Xiang B. Enhanced microbiota profiling in patients with quiescent Crohn’s disease through comparison with paired healthy first-degree relatives. Cell Rep Med. (2024) 5(7):101624. doi: 10.1101/2024.02.24.581863, PMID: 38942021 PMC11293350

[54] Wilson BC Vatanen T Cutfield WS O’Sullivan JM. The super-donor phenomenon in fecal microbiota transplantation. Front Cell Infect Microbiol. (2019) 9:430737. doi: 10.3389/fcimb.2019.00002, PMID: 30719428 PMC6348388

[55] Porcari S Benech N Valles-Colomer M Segata N Gasbarrini A Cammarota G. Key determinants of success in fecal microbiota transplantation: From microbiome to clinic. Cell Host Microbe. (2023) 31:712–33. doi: 10.1016/j.chom.2023.03.020, PMID: 37167953

[56] Reddi S Senyshyn L Ebadi M Podlesny D Minot SS Gooley T. Fecal microbiota transplantation to prevent acute graft-versus-host disease: pre-planned interim analysis of donor effect. Nat Commun. (2025) 16:1–13. doi: 10.1038/s41467-025-56375-y, PMID: 39863610 PMC11762788

[57] El-Salhy M Hatlebakk JG Gilja OH Bråthen Kristoffersen A Hausken T. Efficacy of faecal microbiota transplantation for patients with irritable bowel syndrome in a randomised, double-blind, placebo-controlled study. Gut. (2020) 69(5):859–67. doi: 10.1136/gutjnl-2019-319630, PMID: 31852769 PMC7229896

[58] Ney L-M Wipplinger M Grossmann M Engert N Wegner VD Mosig AS. Short chain fatty acids: key regulators of the local and systemic immune response in inflammatory diseases and infections. Open Biol. (2023) 13:230014. doi: 10.1098/rsob.230014, PMID: 36977462 PMC10049789

[59] FanJWuYWangXUllahHLingZLiuP. The probiotic enhances donor microbiota stability and improves the efficacy of fecal microbiota transplantation for treating colitis. J Adv Res. (2025) S2090-1232(25):00177-8. doi: 10.1016/j.jare.2025.03.017, PMID: 40089059

[60] Cammarota G Ianiro G Tilg H Rajilić-Stojanović M Kump P Satokari R. European consensus conference on faecal microbiota transplantation in clinical practice. Gut. (2017) 66:569–80. doi: 10.1136/gutjnl-2016-313017, PMID: 28087657 PMC5529972

[61] David LA Maurice CF Carmody RN Gootenberg DB Button JE Wolfe BE. Diet rapidly and reproducibly alters the human gut microbiome. Nature. (2014) 505(7484):559–63. doi: 10.1038/nature12820, PMID: 24336217 PMC3957428

[62] He R Li P Wang J Cui B Zhang F Zhao F. The interplay of gut microbiota between donors and recipients determines the efficacy of fecal microbiota transplantation. Gut Microbes. (2022) 14(1):2100197. doi: 10.1080/19490976.2022.2100197, PMID: 35854629 PMC9302524

[63] Zhang Y Wang S Wang H Cao M Wang M Zhang B. Efficacy of donor-recipient-matched faecal microbiota transplantation in patients with IBS-D: A single-centre, randomized, double-blind placebo-controlled study. Digestion. (2024) 105:457–67. doi: 10.1159/000540420, PMID: 39084197

