# Supplementary material for: Microbiota composition-based donor selection affects FMT efficacy in a murine colitis model
Source: Front Immunol. 2025 Aug 1;16:1635244. doi: 10.3389/fimmu.2025.1635244 (PMC12353733; doi:10.3389/fimmu.2025.1635244)
Supplement: Supplementary Figure 1 — High-function donor FMT exhibits superior efficacy in ameliorating IBD-associated dysbiosis compared to other therapeutic cohorts. (A) Shannon index measuring α-diversity among IBD and treatment groups. (B) Anosim analysis based on Bray-Curtis distance. (C) Phylum-level taxonomic composition. (D) Genus-level taxonomic composition. Experimental cohorts: Control, model, H-A, H-B, H-C, H-D, L-A, L-B, L-C, L-D, and 5-ASA group (11 groups). [file Image1.pdf]

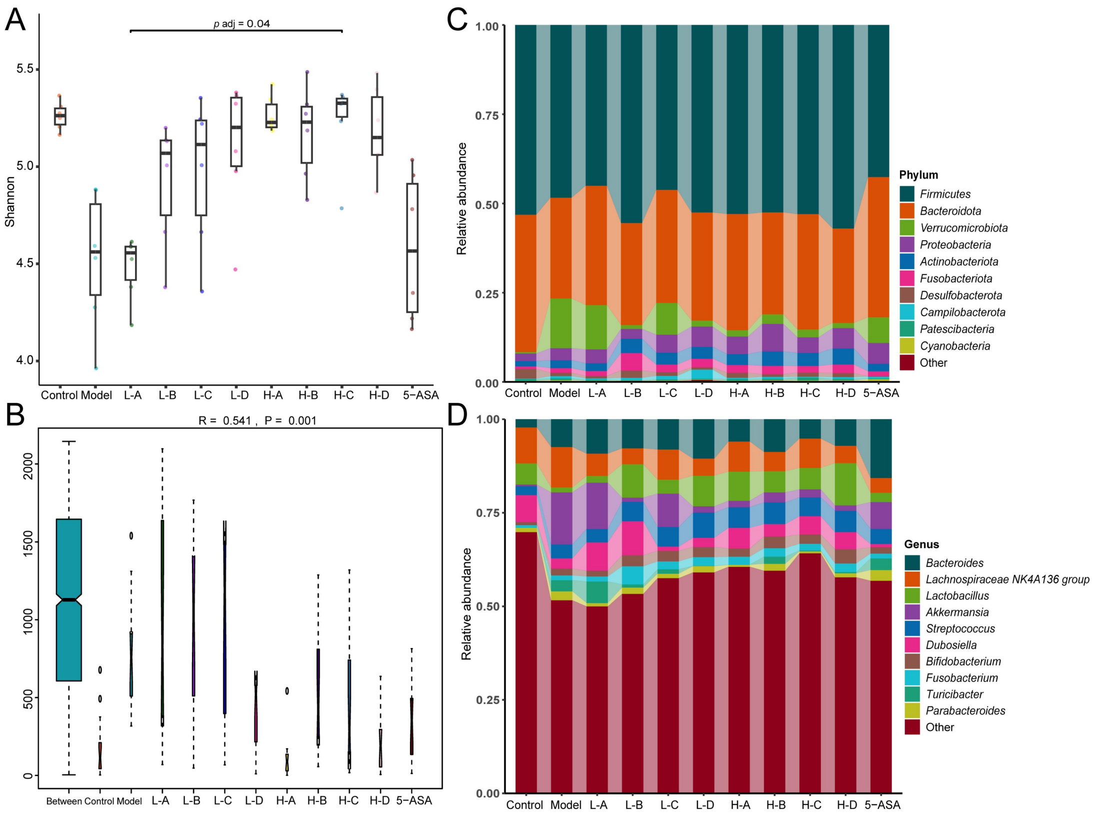

Supplementary Figure 1. High-abundance donor FMT exhibits superior efficacy in ameliorating IBD-associated dysbiosis compared to other therapeutic cohorts. (A) Shannon index measuring α-diversity among IBD and treatment groups. (B) Anosim analysis based on Bray-Curtis distance. (C) Phylum-level taxonomic composition. (D) Genus-level taxonomic composition. Experimental cohorts: Control, model, H-A, H-B, H-C, H-D, L-A, L-B, L-C, L-D, and 5-ASA group (11 groups).
